# Considerations for evaluating the practical utility of machine learning in suicide risk estimation: the role of cost and equity

**DOI:** 10.21203/rs.3.rs-8216032/v1

**Published:** 2025-12-30

**Authors:** Christopher Kitchen, Anas Belouali, Paul S Nestadt, Holly C Wilcox, Hadi Kharrazi

**Affiliations:** Johns Hopkins School of Public Health; Johns Hopkins School of Medicine; Johns Hopkins School of Medicine; Johns Hopkins School of Medicine; Johns Hopkins School of Public Health

## Abstract

A key vulnerability in modeling suicide death is a lack of precision and therefore estimates are thought as ultimately unhelpful to clinicians, even with more advanced or nuanced machine learning (ML) techniques. We sought to fill several conceptual gaps by assessing performance, focusing on the precision-recall tradeoff, across multiple techniques, and with ad hoc contextualization for sensitivity, cost-balance, and fairness. To identify robust, differential performances of a cross section of ML techniques on a suicide risk task, emphasizing overall AUPRC maximization and downstream effects on hypothetical decision support. A retrospective cohort was selected for patients receiving care or having died per the Office of the Medical Examiner (OCME), between 2017 and 2020 using the Maryland Suicide Datawarehouse (MSDW). AUPRC-optimized settings yielded cross-validated AUPRC significantly improved over logistic regressions, especially for XGBoost in both hospital discharge (AUPRC: 0.667; PPV: 0.941) and commercial claims records (AUPRC: 0.558; PPV: 0.857). F-Beta statistics revealed that when precision is preferred (e.g., 99.9 percentile), XGBoost are among the most efficient tools, while random forest and MLP are better when sensitivity is preferred (90 percentile or lower). No algorithmic bias was identified by age, sex or race, but significant changes in performance are noted with certain clinical characteristics. To our knowledge, this is the first use of an AUPRC-maxima optimization for ML tools with predicting suicide death. The utility of suicide risk models in clinical decision support is discussed as being tied to innate class imbalance challenges in model training, with recommendations being provided on how to better evaluate performance.

## BACKGROUND

Suicide risk models hold promise in identifying at-risk patients in large cohorts. [1–4] Lack of practical validity, however, often leads to models performing well in research settings but having limited use in clinical settings. The existing literature on machine learning in suicide risk estimation generally fails to measure and explain the practical validity of new models. This limitation has resulted in reduced clinical applicability of these tools unless they specifically link risk to narrow use cases and cohorts. [5–7]

Suicide risk models tend to be evaluated using area under receiver operating characteristics (AUROC) with some indication of how many correct suicide deaths could be identified out of various sensitivity settings. [1–4, 7, 8] A recent meta-analysis of suicide prediction literature concluded that the wealth of different methods and techniques applied to suicide prediction still falls short of having clinical utility due to lacking precision. [8–12] This lack of precision can be partly due to an improper framing of the risk prediction task as classification rather than event detection. [13, 14]

Multiple factors affect the precision of suicide risk models. Most importantly, suicide risk models are trained on very imbalanced data as models should generally be trained in the same conditions as their eventual use. [15–18] Another reason is that errors in suicide risk prediction are generally treated as equal in, though without considering real-world costs. Additionally, the desire for maximizing sensitivity of such models often result in alert fatigue and providers seeing diminished value in such predictive tools. [1]

Suicide risk models should balance precision (i.e., positive predictive value, or PPV) and false negatives (i.e., sensitivity, or recall). Past research has often addressed the trade-off of precision and recall by comparing risk sensitivity to a threshold (e.g., top 1% or 5% of the response distribution) and then evaluating the corresponding confusion matrix. However, this approach can worsen algorithmic bias in decision support or not generalize well with new data and be an unreliable basis for comparing performance across diverse algorithms. [19, 20] For example, the same 1% may come from an overrepresented group, be highly idiosyncratic, or lead to the wrong conclusion based on outlier cases.

Cost and equity are seldom the focus for suicide risk modeling even though they directly impact the appropriateness of their clinical use. Recent work in the measurement of algorithmic bias has formalized benchmarks for evaluating model performance, making it possible to identify when prospective tools fail a basic fairness test during development. [21–23] Additionally, costs can be calibrated to different balances of precision-recall and summarized using economic impact, confusion matrix or F-beta score. [24–26] However, cost means different concepts to different stakeholders. [24, 25] Advocating for use of one model versus another should be anchored to specific real-world effects (e.g., lives saved, patients screened, dollars spent) during validation. [1, 26]

Suicide risk models are frequently used to identify patients for further screening or intervention; however, these models often lack precision and limited application in clinical context. [9, 11, 12] Using machine learning approaches for suicide risk prediction has also shown improvement in AUPRC, which is critical to the prediction of rare events. [27–30] Nevertheless, using machine learning approaches has further complicated balancing precision and recall while keeping the models fair and cost effective. To address these challenges, this study aimed to: (1) evaluate the circumstances and techniques in which machine learning might be useful, including whether they are robustly better than the more interpretable regression-based models, and (2) compare different approaches to measure clinical utility of risk models, while considering fairness and cost. Results of this study could be used to assess whether the decisions made by the end users of suicide risk models are fair and enhanced for patient outcomes and not just performing adequately for development.

## RESULTS

### Sample Characteristics

The average age of suicide decedents was 49.3 years, making them generally older than either of the living control groups from HSCRC (42.0) or MHCC (40.4) but much younger on average than other decedents from the OCME record (64.4). Suicide decedents were also disproportionately more male (76.9%; χ^2^ = 5157, p < 0.001) and identifying as Caucasian (76.1%; χ^2^ = 7250, p < 0.001) than any other comparison group. Several key psychiatric and behavioral diagnoses were significantly greater among suicide decedents than any other groups, including depressive disorders (χ^2^ = 40036, p < 0.001), anxiety disorders (χ^2^ = 18049, p < 0.001), bipolar disorder (χ^2^ = 14559, p < 0.001), psychotic disorders (χ^2^ = 19006, p < 0.001), post-traumatic stress (χ^2^ = 7771, p < 0.001) and attention-deficit hyperactivity disorder (χ^2^ = 823.24, p < 0.001). Care utilization was noted to be significantly lower among suicide decedents compared to other decedents, but greater than living control groups (Table 1).

### Predictive Modeling

The AUPRC is identified as the principal metric for selecting candidate models for each cohort. For decedents, both elastic net and XGBoost achieved an average, cross-validated AUPRC of 0.251, significantly different from all other performances except logistic regression (0.249). Average AUPRC obtained by XGBoost for HSCRC (0.667, 0.663:0.670) and MHCC (0.558, 0.554:0.563) are significantly greater than all alternative models for those cohorts, based on their respective 95% confidence intervals. HSCRC and MHCC-based XGBoost models also produced the highest PPVs of 0.941 and 0.857 with a classification threshold of 0.5 for response probability (Table 2).

**Figure 1:**
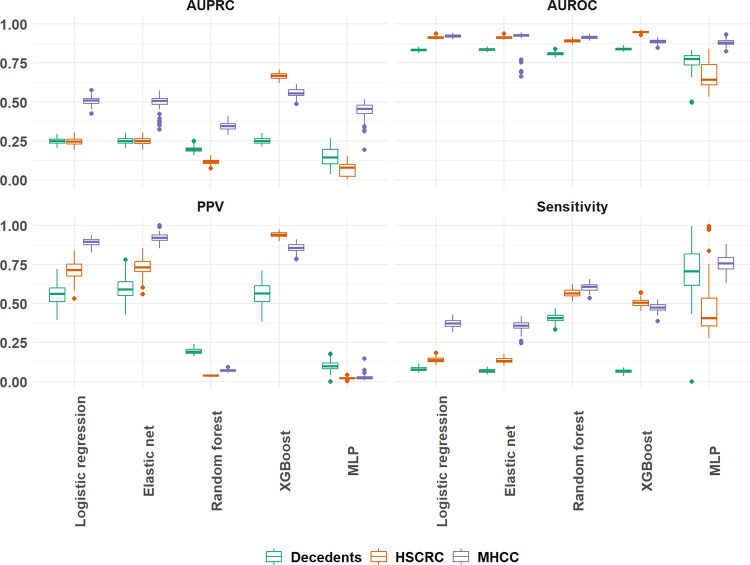
Primary cross-validated model performance by model type and data source.

PPV was compared to optimized AUPRC and sensitivity across all algorithms to assess divergences in performance. Elastic net and regression showed high precision but comparatively low sensitivity across each data source (sensitivity: Decedents 0.067, 0.065:0.069; HSCRC 0.133, 0.130:0.136; MHCC: 0.356, 0.350:0.361). In contrast, random forest models resulted in high sensitivity but at the expense of much lower precision across all cohorts (PPV: Decedents 0.193, 0.190:0.196; HSCRC 0.037, 0.037:0.038; MHCC 0.072, 0.070:0.073). XGBoost models produced higher precision and recall over other estimates, though only for the HSCRC and MHCC cohorts (F1: Decedents 0.115, 0.111:0.118; HSCRC 0.656, 0.652:0.660; MHCC 0.610, 0.606:0.614). Neither ensemble methods nor the MLP were as successful in achieving optimal AUPRC for the HSCRC and MHCC cohorts but resulted in better sensitivity than most alternatives (Fig. 1).

Variable importance for the top 20 most influential features was largely consistent across models for each cohort (**Supplemental Figures S1A-S**1C). More divergences in rank emerged from the elastic net, likely due to the manner it handles collinearity among features by penalized weights [54]. The median rescaled rank of most important features for each cohort tended to converge on age (Median: Decedents 0.998; HSCRC: 0.868; MHCC: 0.955), male sex (Decedents 0.974; HSCRC: 0.976; MHCC: 0.967), prior ideation or attempt (Decedents: 0.972; HSCRC: 0.909; MHCC: 0.966), depression diagnosis (Decedents: 0.881; HSCRC: 0.940; MHCC: 0.938), and hospitalization within 12 months (Decedents: 0.879; HSCRC: 0.847; MHCC: 0.860) (**Supplemental Table S2**).

Classification metrics were measured across multiple thresholds for percentile risk (Table 3). For each cohort, PPV increased as a function of restricting the threshold to higher risk. Conversely, sensitivity was reduced, leaving uncertainty in which threshold is best suited to balancing precision and recall. The best F1 for each algorithm was obtained at the 90th percentile decedents (suicide rate = 0.041), but for HSCRC (rate = 0.002) and MHCC (rate = 0.002) best F1 was achieved at the 99.9th percentile. At these thresholds, XGBoost outperformed most alternatives for precision, with an average 941 cases correctly predicted among decedents (PPV: 0.198; sensitivity: 0.484), 908 in HSCRC (PPV: 0.961; sensitivity: 0.467) and 858 cases in MHCC (PPV: 0.906; sensitivity: 0.441) (Table 3).

### Cost and Fairness of Estimated Risk

Different ad hoc interpretations of performance were obtained through F-beta estimates that reflect a preference for greater precision or recall [24]. At the 90th percentile among decedents, a beta of 0.1 corresponds to weighting precision 10 times over sensitivity and identifies the 99th or 99.9th as better thresholds of classification, across all algorithms. Conversely, for the remaining ‘low rate’ cohorts, valuing sensitivity 10 times over precision generally reduces the preferred threshold from the 99.9th to the 99th percentile, and only for the most precise models (i.e., excluding Random Forest) (Table 3).

The disparate impact of suicide death among subgroupings was consistently imbalanced and less than 0.8 for male or Caucasian patients, and those with psychiatric conditions, outpatient care or social needs (**See Supplemental Figure S2**). Two notable exceptions were age greater than 65 years in MHCC (i.e., a higher rate of suicide death) and having zero outpatient encounters among decedents.

**Figure 2:**
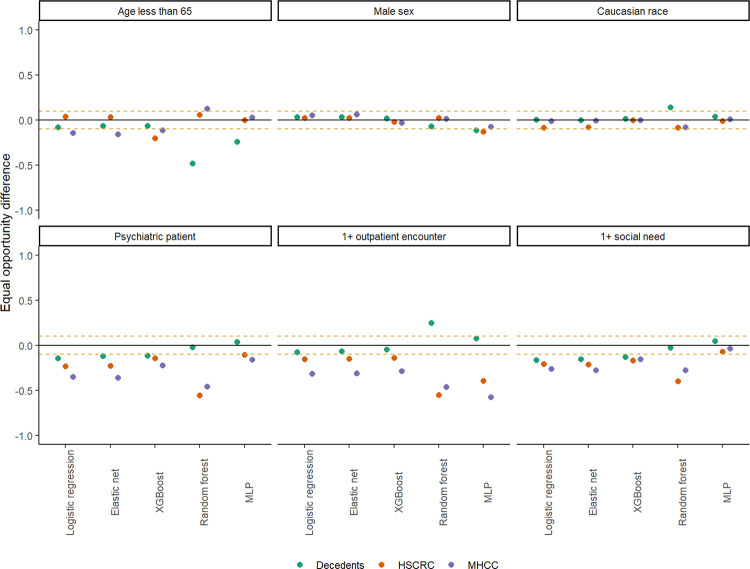
Estimated equal opportunity difference in classification for suicide death, by model type and source of data.

Cross-validated equal odds difference (EOD) was estimated to be fair for most fitted models when patient demographics defined a privileged group in (Fig. 2, **Supplemental Table S4**). Some exceptions were noted among decedents for high sensitivity models (e.g., random forest and MLP). XGBoost was again observed to perform well with these first three demographic strata across each cohort, the range being − 0.203 to −0.066 for age less than 65; −0.032 to 0.020 for male sex; and − 0.086 to 0.013 for Caucasian race. These values were consistent across each algorithm and grouping, though more biased towards younger decedents (i.e., higher predicted odds) when using random forest and MLP. The three clinically defined groups were also more favorable to their respective out-groups. EOD favored correct classifications between − 0.557 to − 0.020 (across all models, cohorts) for patients with psychiatric conditions, with the random forest model generally being the least fair for HSCRC and MHCC records. For those with 1 + outpatient encounter the range was − 0.583 and 0.248, and for 1 + ICD-10-CM coded social need it was − 0.398 and 0.044 with both MLP and random forest methods proving to be among the least equitable (**Supplemental Table S4**).

## DISCUSSION

A key restriction in predicting suicide risk is the lack of precision thus limiting the scalability and practical utility of such models. Cost-weighting suicide risk models are challenging, especially when the consequence of a false negative is patient death, and a monetary value applied to false positives. To address this challenge, this study frames suicide risk modeling as a signal detection task by incorporating AUPRC-maxima optimization. The study hypothesizes that deciding candidate suicide risk models for implementation should depend both on the cross-validated AUPRC and the theoretical cost and fairness for different cohorts of interest.

Similar values for AUROC and classification metrics have been observed for suicide risk prediction models from prior literature, especially where large class imbalance is present. [8] For example, McCarthy et al found ROC, PPV and sensitivity values of 0.90, 0.38 and 0.001, using logistic regression with a threshold setting of the 90th percentile and a cohort where the rate of suicide death was 36 per 100,000 patients. [55] The Belsher et al. meta-analysis generally observed the same benchmark sensitivity statistics for these thresholds with very low associated PPV across all threshold settings (between < 0.001 and .19). Similarly, in this study, for the most imbalanced cohorts of HSCRC and MHCC, results showed an analogous tradeoff of precision and recall (Table 2), and no value for interpreting ROC for logistic regression at the 90th percentile (i.e., 0.913 and 0.922 for ROC, 0.016 and 0.017 for PPV, 0.766 and 0.810 for sensitivity, using HSCRC and MHCC cohorts respectively) (Table 3). However, classifying the top 10% of patients using this approach identifies around 95,000 individuals as at risk, which are too many patients to intervene on and the reason for distorted PPV estimates.

Results of this study differ in that the candidate machine learning model, AUPRC-optimized XGBoost, was associated with the best average AUPRC in each of the full samples (Decedents: 0.251; HSCRC: 0.667; MHCC: 0.558) (Table 3). XGBoost also showed a significantly greater performance in the most imbalanced cohorts. By raising the classification threshold and using AUPRC-optimization of hyperparameters, it was possible to obtain a better overall balance between precision and recall, and enhanced precision. At the 99.9th percentile, XGBoost obtained a cross-validated F1 of 0.629 and 0.594, PPV of 0.961 and 0.906 and sensitivity of 0.467 and 0.441, for HSCRC and MHCC respectively (Table 3). For the less imbalanced decedents cohort, XGBoost was again among the top performing models for AUPRC but fell behind random forest classification metrics (Fig. 1).

Rates of suicide are unequal across different clinical cohorts. This study reported on the disparate impact of suicide death from these samples but also demonstrate that the EOD of XGBoost models tended to yield largely unbiased responses for age, sex and race (Fig. 2). Better estimation of suicide risk was generally possible when clinical groups for psychiatric diagnosis, outpatient care utilization, and social needs were present (**Supplemental Table S4**). These findings could be partially due to missingness of clinical information, often through limited healthcare utilization or access. Consequently, this could lead to training biased models that detect high risk cases among clinically vulnerable populations and not the population at large. Thus, a one-size-fits-all approach to estimate suicide risk seems to be inappropriate, and separate models for points of care or clinical composition may be best for the task.

A way to separate risk estimation from the degree of class imbalance might be to focus on cost weighting or the F-beta classification score. Critically, these values illustrate how the preferred candidate model changes as a function of differentially weighting precision and recall. For example, the random forest model was seen to yield the best classification performance when recall was 10 times preferred over precision among decedents, but not when greater precision was needed (e.g., F0.1 versus F10 score). In highly imbalanced data, the XGBoost and weighted ensemble models performed well across all betas except those representing the greatest preference for sensitivity (Table 3).

This study provides context around the sentiment shared in a prior meta-analysis, that in suicide risk modeling “precision is not adequate to be useful.” [9] Although this statement can be true, this could merely be a consequence of the threshold settings and class imbalance found in each study included in the meta-analysis. This study showed that when the threshold is restricted to a small proportion of the cohort (i.e., very high percentiles) or class imbalance is left to something resembling the epidemiological rate of suicide (i.e., no resampling), it is still possible to calibrate useful machine learning tools by using parameters that maximize AUPRC. Additionally, maximizing AUPRC does not appear to drastically affect the theoretical fairness of these models, even though the lack of clinical information does.

Findings of this study do not replace the need for rigorous external validation using new data sources and randomized samples for testing decision support, nor does it replace eventual randomized trials for such applications. The explored methodology merely illustrates how downstream considerations of cost and fairness might be addressed as part of the development of risk models, particularly those with severe class imbalance. The performance estimates, though robustly cross-validated, are not necessarily a firm indicator of the generalizability of findings, nor are the variable importance estimates necessarily correct estimates of individual risk factors.

This study has a few limitations. First, the experiments only used portions of MSDW, which may have inadvertently contributed to a degree bias in the results. The MHCC record, for example, consists of commercial claims records, and so tends to represent employer insured individuals of working age, rather than Medicare and Medicaid beneficiaries. Second, the study limited the identification of patients with social needs through ICD-10-CM coding alone. Many have limited access to care or are uninsured, making it more uncertain whether algorithmic bias was adequately avoided. Finally, the proposed methodology does not address the common sampling techniques used to adjust class imbalance in training. Prior work has shown that relying on such techniques as under/oversampling cases tend to negatively bias AUPRC performance and inflate classification sensitivity at the cost of precision. [56]

Suicide risk estimation benefits from a careful understanding of how clinical decisions are to be supported. A highly sensitive model can be calibrated out of a diversity of ML techniques, and similarly a high-precision tool can be achieved if that is instead desired. Few studies to date have explored model efficiency, fairness and cost. Clinical composition and intended use, dictate which tools are best suited for each task, but our findings suggest a generalizable risk model might be best achieved through use of extreme gradient boosted trees in conditions of significant class imbalance.

## METHODS

### Participants and setting

Observational data from the Maryland Suicide Data Warehouse (MSDW) were used in this study. MSDW is a repository of administrative records, electronic health records (EHRs), and claims data sources, spanning 2012 to 2020. MSDW contains 104,516 decedents linked to the state Office of the Chief Medical Examiner (OCME) records. [31–33] MSDW also contains records from 6,658,990 living patients largely from hospital discharges of the Health Services Cost Review Commission (HSCRC) and commercial claims of the Maryland Health Care Commission (MHCC). [35, 36] All MSDW records have been deidentified by their constituent providers and required by respective data use agreements. Use of MSDW for this project was reviewed and approved by the Johns Hopkins Bloomberg School of Public Health Institutional Review Board. All work was conducted according to relevant guidelines and regulations concerning human subject designs, using retrospective data. This research was approved as minimal risk and informed consent was waived given the risk to participants being reidentified.

The study population included 5,059 decedents from MSDW whom had been identified as having died in a manner consistent with suicide. The study population also included three control groups from MSDW to assess the performance of suicide risk models: (1) Decedents identified by the OCME who have died in a manner other than suicide; (2) Living population represented by the statewide HSCRC hospital discharge data source (capped at 1 million random sample); and, (3) Living population represented by the statewide MHCC administrative claims data (capped at 1 million random sample). These controls were selected to simulate actual use cases by state level stakeholders such as the ME, HSCRC or MHCC. For example, models for classifying manner of death may be useful for identifying probable cases for psychiatric autopsy and OCME review. [34, 35]

Additional inclusion criteria included having a non-missing and valid value for age, sex, and residential address (i.e., Census tract or 5-digit zip code) within the state of Maryland; and having at least one clinical encounter between 2016 and 2020. Decedents were required to have had a date of death 2017 or later. After applying these criteria, 47,529 (45.5% selected) ME-identified decedents were found in the HSCRC or MHCC records, 1,944 (4.1%) of whom died by suicide. Total of 846,542 (84.7%) living patients remained from the HSCRC (hospital discharge) records and 844,331 (84.4%) living patients from the MHCC (administrative claims) records. [36, 37]

### Study design

A retrospective cohort analysis was employed to construct the study population. The study used validated cases of suicide death linked across several data sources, which has been often missing in prior research. [32] Performance optimization was conducted using the AUPRC as a benchmark for comparison in addition to using a competing model framework for assessing risk of suicide. Optimization using AUPRC was applied to decrease the failure of models to generalize properly to new sources of data due to differences in case balance during training and validation. [13–16, 38]

### Variable definitions

The MSDW aggregates observed values from multiple sources on patient demographics (age/sex), marital status and residency. Many clinical indicators were identified in the MSDW using diagnostic categories from the 2020 Clinical Classifications Software, published by the Agency for Healthcare Research and Quality (AHRQ). [39–41] The number of diagnostic and procedure-coded observations identified using this approach was generally limited to chronic diseases or known correlates of suicide risk, substance use disorders, and socio-behavioral needs as supported by literature and independent expert review by two licensed psychiatrists.[42] The Charlson comorbidity score and associated clinical markers were also calculated using diagnostic codes. [43] Pharmacological classes were attached to records for which pharmacy records exist (i.e., MHCC), using the openFDA programming interface and linkage through 11-digit national drug code (NDC). [44] Patient residency information from the OCME and HSCRC records at Census tract level were clustered using the SaTScan procedure for identifying regions with significantly greater than expected suicide cases. [45] This was included to control geographic effects for each model, though only available by Census tract, which precluded its use in MHCC.

Clinical and geographic variables were discretized for a period of observation beginning January 1st, 2016, and ending 7 days prior to recorded date of death for decedents, or a randomly chosen date between 2017 and 2020 for living controls. Each patient had at least 1 year of observation recorded and 1 clinical encounter throughout the full observation period as a result. Additional features reflect the counts of clinical encounters at different points of care within 12 months of each patient’s index date.

A total of 180 variables were identified across the sources of data (i.e., OCME, HSCRC and MHCC). An additional 531 variables were created as interactions between select diagnostic categories and factors known to be associated with suicide risk in observational research. These factors included depressive diagnoses, anxiety, bipolar, psychotic disorders, attention-deficit hyperactivity disorder, post-traumatic stress, opiate or alcohol use disorder, and suicide ideation or attempt. Least absolute shrinkage and selection (LASSO) were applied using the decedents cohort to narrow down the final features to 220 variables (HSCRC:172, MHCC:180). LASSO is frequently used in machine learning workflows to reduce collinearity of inputs and select features. [4, 8]

### Statistical analysis

The main findings of this analysis are centered on the evaluation of AUPRC-optimized suicide risk models in a cross-validation framework. Sampling consisted of repeated 5-fold cross-validation with 20 iterations, for a total distribution of performance metrics on 100 model fits to evaluate uncertainty. Average performance metrics that exceed or fall below the 95% confidence interval of comparison distributions were considered significantly different for the purpose of understanding the value of using algorithm over another.

Multiple discrete approaches were considered for predicting suicide risk: two regression-based models (i.e., logistic regression and elastic net regression) were contrasted with two decision tree ensemble methods (i.e., random forest and extreme gradient boosted trees, i.e., XGBoost) to contrast methods that have underlying assumptions about these data. Finally, a separate multilayer perceptron (MLP) was was added to test whether complex associations between features and suicide could be accounted for and imrove classification. [9, 45] All analyses were conducted using the R programming language (version 4.0.2). [47–53]

Optimization involved a single 5-fold cross validation for each of the chosen hyperparameters in a truncated grid search. Hyperparameters were considered optimal when average AUPRC was highest for each cohort and algorithm pairing. Full ranges of performance (i.e., dispersion per setting) as well as inspection of local maxima were part of this process, but the chosen parameters were generally univariate on their impact in performance, except for learning rate (η). Tested parameters included (a) alpha and lambda-solution (maximum AUROC, minimized error and 1 standard deviation below minimized error) for elastic net, (b) depth, number of trees and positive class weight for random forest, (c) depth, number of trees and learning rate for extreme gradient boosting (XGBoost), and (d) learning rate, dropout rate and batch size for 3-layer perceptrons using a ReLU activation and a structure of 128, 64 and 32 hidden nodes. Selected parameters are summarized in the supplemental material (**Supplemental Table 1**).

The interpretability of model effects was limited due to incomparable indices of variable importance. These were algorithm-specific and different between regression and decision tree methods. Average standardized t-values (significant at 0.001 alpha level) and penalized parameters of elastic net were used to indicate variable importance in regressions and mean cross-entropy for both random forest and XGBoost. All measures of variable importance were converted to a ranked value for total remaining attributes in each model and rescaled to a range of zero to one, which facilitated comparison. A list of 5 of the most important features were identified across all models and tabulated with their respective 95% confidence interval (CI). Additionally, importance scores of the top 20 features of the models were plotted for comparison for each data source.

Candidate models were selected based on cross-validated AUPRC and a separate ad hoc cost-fairness analysis. Responses were limited to 1/10th of the total number of CV iterations. Cost was explored as a calibration of F-beta at different threshold settings, indexing classification by the relative value of precision to sensitivity. [24, 25] The F1 score was reported with other classification metrics and represents equal weight being applied to precision and sensitivity. Six demographic and clinical strata were identified as group characteristics to address algorithmic bias. Average cross-validated equal opportunity difference (EOD) was reported across all cross-validated performances and considered fair if falling between − 0.1 and 0.1. [21–23] The disparate impact of suicide death was reported for each strata.

## Supplementary Material

Supplementary Files

This is a list of supplementary files associated with this preprint. Click to download.

- FigureS1A.png
- FigureS1B.png
- FigureS1C.png
- figureS2.png
- SupplementalMaterials.docx

## Figures and Tables

**Table 1:** Patient characteristics across each data source compared with suicide decedents.

Attribute	Non-suicide decedents	HSCRC (living patients)	MHCC (living patients)	Suicide decedents	X^2^/F-stat**
Total patients	45,585	846,542	844,331	1,944	
Average age	m = 64.4 (sd = 19.7)	m = 42.0 (sd = 23.5)	m = 40.4 (sd = 22.8)	m = 49.3 (sd = 19.5)	F = 23168*
Female	18,561 (40.7%)	466,373 (55.1%)	440,242 (52.1%)	449 (23.1%)	5156.5*
Male	27,024 (59.3%)	380,169 (44.9%)	404,089 (47.9%)	1,495 (76.9%)	
Caucasian	26,983 (59.2%)	454,857 (53.7%)	138,126 (16.4%)	1,479 (76.1%)	7250.1*
African American	16,641 (36.5%)	290,035 (34.3%)	79,936 (9.5%)	325 (16.7%)	
Other race/ethnicity	1,741 (3.8%)	151,806 (17.9%)	51,291 (6.1%)	135 (6.9%)	
Missing race/ethnicity	220 (0.5%)	16,406 (1.9%)	586,228 (69.4%)	5 (0.3%)	875299*
Depressive disorders	16,637 (36.5%)	95,062 (11.2%)	69,595 (8.2%)	826 (42.5%)	40036*
Anxiety disorders	13,734 (30.1%)	107,402 (12.7%)	86,339 (10.2%)	671 (34.5%)	18049*
Bipolar disorders	4,179 (9.2%)	20,263 (2.4%)	11,836 (1.4%)	254 (13.1%)	14559*
Psychotic disorders	3,314 (7.3%)	10,581 (1.2%)	5,159 (0.6%)	176 (9.1%)	19006*
Post-traumatic stress	4,173 (9.2%)	22,198 (2.6%)	34,569 (4.1%)	281 (14.5%)	7771.1*
Attention-deficit hyperactivity	1,740 (3.8%)	18,548 (2.2%)	20,932 (2.5%)	153 (7.9%)	823.24*
Suicide ideation or attempt	3,400 (7.5%)	21,060 (2.5%)	192 (0%)	438 (22.5%)	36144*
Alcohol use disorder	7,703 (16.9%)	31,044 (3.7%)	10,316 (1.2%)	350 (18%)	44344*
Opiate use disorder	6,629 (14.5%)	15,025 (1.8%)	5,201 (0.6%)	209 (10.8%)	56364*
Cannabis use disorder	4,426 (9.7%)	27,748 (3.3%)	7,070 (0.8%)	220 (11.3%)	23775*
Charlson comorbidity index	m = 3.9 (sd = 4.3)	m = 0.7 (sd = 1.8)	m = 0.7 (sd = 2.4)	m = 1.5 (sd = 2.9)	F = 38288*
1 + hospitalization	27,071 (59.4%)	94,517 (11.2%)	44,831 (5.3%)	734 (37.8%)	152051*
1 + emergency encounter	25,231 (55.3%)	58,816 (6.9%)	38,795 (4.6%)	644 (33.1%)	170875*
1 + outpatient encounter	40,273 (88.3%)	550,999 (65.1%)	404,907 (48%)	1,632 (84%)	69674*
1 + psychiatric hospitalization	616 (1.4%)	1,630 (0.2%)	118 (0%)	66 (3.4%)	7395*

* Significant at p < 0.001

** Chi-squared tests of independence for each attribute (one-way ANOVA for continuous attributes)

**Table 2:** Average (95% CI) cross-validated performance of each model by source of data and metric.

Cohort	Model*	AUROC	AUPRC	F1	Sensitivity	Specificity	PPV
OCME Decedents	Elastic net	0.837 (0.835:0.838)	0.251 (0.248:0.255)	0.12 (0.116:0.123)	0.067 (0.065:0.069)	0.998 (0.998:0.998)	0.593 (0.580:0.606)
	Logistic regression	0.833 (0.832:0.835)	0.249 (0.245:0.252)	0.139 (0.136:0.143)	0.08 (0.077:0.082)	0.997 (0.997:0.997)	0.559 (0.547:0.57)
	MLP	0.746 (0.730:0.761)	0.145 (0.135:0.155)	0.165 (0.154:0.176)	0.661 (0.62:0.702)	0.735 (0.709:0.761)	0.096 (0.089:0.103)
	Random forest	0.808 (0.807:0.810)	0.196 (0.193:0.199)	0.261 (0.258:0.264)	0.407 (0.402:0.411)	0.927 (0.926:0.929)	0.193 (0.19:0.196)
	XGBoost	0.840 (0.839:0.842)	0.251 (0.248:0.255)	0.115 (0.111:0.118)	0.064 (0.062:0.066)	0.998 (0.998:0.998)	0.564 (0.551:0.576)
HSCRC *(Hospital* *Discharges)*	Elastic net	0.914 (0.913:0.916)	0.249 (0.244:0.254)	0.225 (0.22:0.23)	0.133 (0.13:0.136)	0.999 (0.999:0.999)	0.733 (0.722:0.743)
	Logistic regression	0.913 (0.912:0.915)	0.247 (0.242:0.251)	0.232 (0.227:0.238)	0.139 (0.136:0.143)	0.999 (0.999:0.999)	0.713 (0.702:0.724)
	MLP	0.666 (0.653:0.679)	0.068 (0.06:0.075)	0.038 (0.036:0.04)	0.459 (0.432:0.486)	0.919 (0.892:0.947)	0.02 (0.019:0.021)
	Random forest	0.891 (0.889:0.893)	0.116 (0.113:0.119)	0.07 (0.069:0.071)	0.566 (0.561:0.571)	0.966 (0.966:0.967)	0.037 (0.037:0.038)
	XGBoost	0.949 (0.948:0.95)	0.667 (0.663:0.67)	0.656 (0.652:0.66)	0.504 (0.499:0.509)	0.999 (0.999:0.999)	0.941 (0.938:0.944)
MHCC *(Claims* *Data)*	Elastic net	0.914 (0.904:0.924)	0.498 (0.49:0.505)	0.512 (0.506:0.518)	0.356 (0.35:0.361)	0.999 (0.999:0.999)	0.921 (0.915:0.926)
	Logistic regression	0.922 (0.92:0.924)	0.507 (0.503:0.512)	0.525 (0.52:0.529)	0.372 (0.368:0.377)	0.999 (0.999:0.999)	0.891 (0.886:0.895)
	MLP	0.882 (0.878:0.885)	0.445 (0.435:0.454)	0.047 (0.042:0.053)	0.758 (0.748:0.768)	0.907 (0.897:0.916)	0.025 (0.022:0.028)
	Random forest	0.914 (0.913:0.916)	0.346 (0.342:0.351)	0.128 (0.126:0.13)	0.604 (0.599:0.608)	0.982 (0.982:0.982)	0.072 (0.07:0.073)
	XGBoost	0.886 (0.884:0.888)	0.558 (0.554:0.563)	0.61 (0.606:0.614)	0.474 (0.469:0.479)	0.999 (0.999:0.999)	0.857 (0.852:0.862)

* Models are sorted alphabetically

**Table 3:** Estimated test-performance using a 20% hold out sample, with corresponding sensitivity settings for percentile rank of risk, and associated F-betas.

Decedents				Classification metric				F-beta				
Algorithm	**%tile**	**AUROC**	**AUPRC**	**Acc.**	**Sens.**	**Spec.**	**PPV**	**0.1**	**0.5**	**1**	**2**	**10**
Elastic net	90th	0.837	0.251	0.899	0.488	0.917	0.200	0.201	0.226	0.283	0.379	0.481
	99th			0.959	0.118	0.995	0.481	0.467	0.298	0.189	0.139	0.119
	99.9th			0.960	0.019	0.999	0.750	0.539	0.084	0.036	0.023	0.019
Logistic regression	90th	0.833	0.249	0.899	0.484	0.916	0.198	0.199	0.225	0.281	0.376	0.477
	99th			0.959	0.118	0.995	0.483	0.469	0.299	0.190	0.139	0.119
	99.9th			0.960	0.019	0.999	0.770	0.554	0.087	0.037	0.024	0.019
MLP	90th	0.746	0.145	0.899	0.357	0.922	0.162	0.163	0.182	0.223	0.288	0.352
	99th			0.958	0.044	0.997	0.357	0.334	0.148	0.079	0.053	0.044
	99.9th			0.959	0.013	0.999	0.547	0.393	0.061	0.026	0.017	0.014
Random forest	90th	0.808	0.196	0.896	0.439	0.916	0.182	0.183	0.206	0.257	0.342	0.433
	99th			0.956	0.086	0.993	0.356	0.345	0.219	0.139	0.102	0.087
	99.9th			0.959	0.014	0.999	0.643	0.444	0.064	0.027	0.017	0.014
XGBoost	90th	0.840	0.251	0.899	0.484	0.916	0.198	0.199	0.225	0.281	0.376	0.477
	99th			0.959	0.117	0.995	0.477	0.463	0.295	0.188	0.138	0.118
	99.9th			0.960	0.019	0.999	0.750	0.539	0.084	0.036	0.023	0.019
HSCRC				**Classification metric**				**F-beta**				
Algorithm	**%tile**	**AUROC**	**AUPRC**	**Acc.**	**Sens.**	**Spec.**	**PPV**	**0.1**	**0.5**	**1**	**2**	**10**
Elastic net	90th	0.914	0.249	0.901	0.771	0.901	0.016	0.016	0.020	0.031	0.073	0.524
	99th			0.990	0.456	0.991	0.094	0.095	0.112	0.156	0.258	0.439
	99.9th			0.998	0.213	0.999	0.438	0.434	0.362	0.287	0.237	0.214
Logistic regression	90th	0.913	0.247	0.901	0.766	0.901	0.016	0.016	0.020	0.031	0.073	0.521
	99th			0.990	0.451	0.991	0.093	0.093	0.110	0.154	0.254	0.434
	99.9th			0.998	0.210	0.999	0.432	0.427	0.356	0.282	0.234	0.211
MLP	90th	0.666	0.068	0.900	0.506	0.901	0.010	0.011	0.013	0.020	0.048	0.344
	99th			0.992	0.197	0.994	0.061	0.061	0.071	0.093	0.136	0.193
	99.9th			0.997	0.101	0.999	0.208	0.206	0.172	0.136	0.113	0.102
Random forest	90th	0.891	0.116	0.903	0.709	0.903	0.015	0.015	0.019	0.029	0.069	0.485
	99th			0.990	0.414	0.991	0.085	0.086	0.102	0.142	0.234	0.399
	99.9th			0.998	0.128	0.999	0.287	0.283	0.229	0.177	0.144	0.128
XGBoost	90th	0.949	0.667	0.902	0.871	0.902	0.018	0.018	0.022	0.035	0.083	0.592
	99th			0.991	0.752	0.992	0.155	0.156	0.184	0.257	0.424	0.724
	99.9th			0.999	0.467	0.9999	0.961	0.951	0.793	0.629	0.521	0.469
MHCC				**Classification metric**				**F-beta**				
Algorithm	**%tile**	**AUROC**	**AUPRC**	**Acc.**	**Sens.**	**Spec.**	**PPV**	**0.1**	**0.5**	**1**	**2**	**10**
Elastic net	90th	0.914	0.498	0.901	0.817	0.901	0.017	0.017	0.021	0.033	0.078	0.555
	99th			0.991	0.634	0.991	0.130	0.131	0.155	0.216	0.358	0.611
	99.9th			0.999	0.395	0.999	0.810	0.802	0.669	0.531	0.440	0.397
Logistic regression	90th	0.922	0.507	0.901	0.8097	0.901	0.017	0.017	0.021	0.033	0.077	0.550
	99th			0.991	0.630	0.991	0.129	0.130	0.154	0.215	0.355	0.607
	99.9th			0.999	0.391	0.999	0.803	0.794	0.663	0.526	0.436	0.393
MLP	90th	0.882	0.445	0.901	0.767	0.901	0.016	0.016	0.020	0.031	0.073	0.521
	99th			0.990	0.598	0.991	0.122	0.123	0.146	0.203	0.337	0.576
	99.9th			0.998	0.373	0.999	0.766	0.758	0.633	0.502	0.416	0.375
Random forest	90th	0.914	0.346	0.902	0.814	0.902	0.017	0.017	0.021	0.033	0.078	0.555
	99th			0.990	0.514	0.991	0.106	0.107	0.126	0.175	0.290	0.495
	99.9th			0.998	0.277	0.999	0.579	0.573	0.474	0.373	0.307	0.276
XGBoost	90th	0.886	0.558	0.901	0.754	0.901	0.015	0.016	0.019	0.030	0.072	0.512
	99th			0.991	0.641	0.991	0.132	0.133	0.157	0.218	0.361	0.617
	99.9th			0.999	0.441	0.999	0.906	0.897	0.748	0.594	0.492	0.444

## Data Availability

These data are subject to multiple data use agreements from different agencies, and though fully deidentified we are prohibited from sharing repurposed data without prior authorization, per the terms of these agreements. On request, additional information in aggregate may be provided from the MSDW or our related work.
